# AEG-1 activates Wnt/PCP signaling to promote metastasis in tongue squamous cell carcinoma

**DOI:** 10.18632/oncotarget.6573

**Published:** 2015-12-12

**Authors:** Yunping Pan, Xu Guo, Zheng Yang, Shan Chen, Yiyan Lei, Millicent Lin, Liantang Wang, Chongjin Feng, Zunfu Ke

**Affiliations:** ^1^ Department of Stomatology, the First Affiliated Hospital, Sun Yat-sen University, Guangzhou 510080, Province Guangdong, P.R.China; ^2^ Department of Pathology, the First Affiliated Hospital, Sun Yat-sen University, Guangzhou 510080, Province Guangdong, P.R.China; ^3^ Department of Gynecology, the First Affiliated Hospital, Sun Yat-sen University, Guangzhou 510080, Province Guangdong, P.R. China; ^4^ Department of Chest Surgery, the First Affiliated Hospital, Sun Yat-sen University, Guangzhou 510080, Province Guangdong, P.R.China; ^5^ Department of Molecular and Medical Pharmacology, Crump Institute for Molecular Imaging (CIMI), California NanoSystems Institute (CNSI), University of California, Los Angeles, California, USA

**Keywords:** AEG-1, epithelial-mesenchymal transition, tongue squamous cell carcinoma, Wnt/PCP, metastasis

## Abstract

Despite advances in therapy, survival among patients with locally advanced squamous cell carcinoma of tongue (TSCC) and cervical lymph node metastasis remains dismal. Here, we estimated the functional effect of AEG-1 on TSCC metastasis and explored the molecular mechanism by which AEG-1 stimulates epithelial-mesenchymal transition (EMT). We initially found that AEG-1 mRNA levels were much higher in metastatic TSCC than in non-metastatic TSCC and that AEG-1 expression strongly correlates with EMT status. Receiver operating characteristic analysis showed that the combined AEG-1 and EMT statuses are predictive of the survival rate among TSCC patients. In addition, AEG-1 knockdown inhibited EMT in cultured TSCC cell lines and in a xenograft-mouse model. Recombinant AEG-1 activated Wnt/PCP-Rho signaling, and its stimulatory effects on TSCC cell invasiveness and EMT were reversed by an anti-Wnt5a neutralizing antibody or by inhibition of Rac1 or ROCK. These results highlight the critical stimulatory effect of AEG-1 on cancer cell invasiveness and EMT and indicate that AEG-1 may be a useful prognostic biomarker for TSCC patients.

## INTRODUCTION

Despite advances in treatment strategies that entail combinations of surgery, chemotherapy and radiotherapy, overall survival among patients with squamous cell carcinoma of tongue (TSCC) with locally advanced disease and cervical lymph node metastasis remains dismal [1]. There is thus a critical need to clarify the underlying molecular mechanisms involved in TSCC invasion and metastasis.

Epithelial-mesenchymal transition (EMT) is a physiological process whereby epithelial cells acquire the motile and invasive phenotypes of mesenchymal cells [2]. EMT is crucial to the acquisition of metastatic potential in many cancers, including TSCC [3]. Early during metastatic progression, transcription factors such as Snail, Slug, Twist, Goosecoid and ZEB2 act directly or indirectly to regulate EMT-associated genes and induce EMT, which is characterized by the loss of cell-cell adhesion molecules such as E-cadherin and the gain of mesenchymal markers such as vimentin and fibronectin [4–7]. However, there is little specific information about the molecular processes that govern EMT in TSCC.

Astrocyte elevated gene-1 (AEG-1; also known as metadherin (MTDH)) was first identified as a human immunodeficiency virus (HIV)-1- and tumor necrosis factor (TNF)-α-inducible late response gene in human fetal astrocytes [8]. AEG-1 is now attracting attention from oncologists because of its functional roles in several aspects of tumor progression, including invasion and metastasis [9]. In addition, AEG-1 activates multiple pro-tumorigenic signaling molecules and transduction pathways, including Ha-ras, nuclear factor-kappaB (NF-κB), PI3K/Akt and Wnt/β-catenin [10–12], as well as various signal factors involved in mediating EMT, including TGF-β [13], Wnt [14, 15], Notch [16] and hedgehog [17]. In the present study, we show that silencing AEG-1 suppresses EMT progression in TSCC and reduces cell migration and invasion mediated via the Wnt/PCP signaling pathway. Thus, targeting AEG-1 may suppress EMT at multiple stages and may provide a promising approach to the treatment of TSCC.

## RESULTS

### AEG-1 enhances metastatic ability of TSCC cells

**Figure 1:**
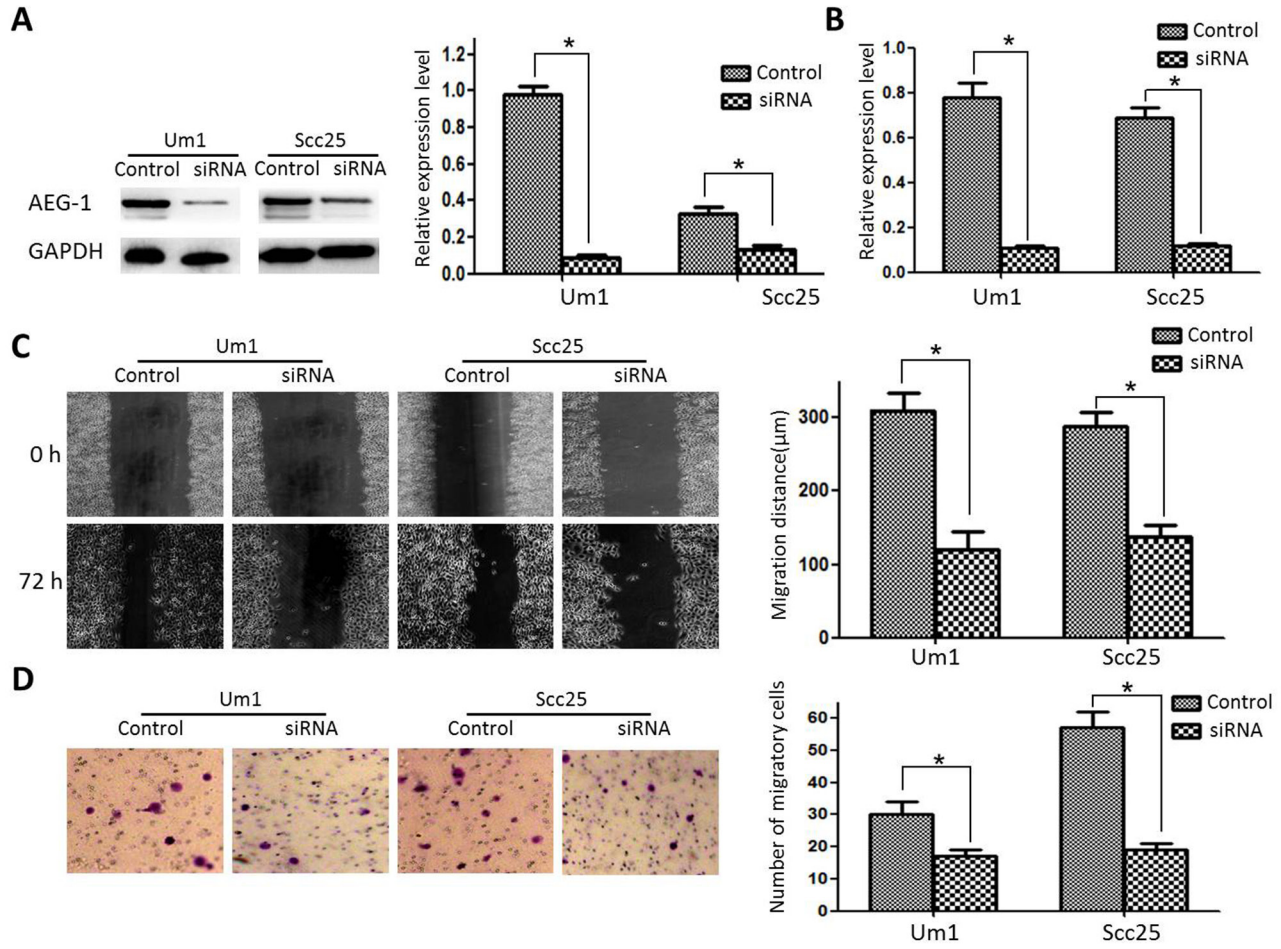
Effect of AEG-1 on metastatic capacity of TSCC cell lines **A.** AEG-1 protein expression in control and knockdown cells. **B.** AEG-1 mRNA expression in control and knockdown cells. **C.** Scratch/wound healing assay revealed that AEG-1 knockdown significantly reduced migration of Um1 and Scc25 cells. **D.** Two-chamber migration assay showed that far fewer AEG-1-siRNA-transfected cells migrated through a Transwell filter.

To examine the effect of AEG-1 on TSCC metastasis, we transfected Um1 and Scc25 cells with a GV248-AEG-1-siRNA to establish stable Um1-siRNA and Scc25-siRNA cell lines in which AEG-1 expression was greatly knocked down compared to the parental cells (Figure 1A and 1B). Subsequent wound-healing assays showed that the control cells migrated approximately 2.8-fold and 2.4-fold farther than Um1-si-AEG-1 and Scc25-si-AEG-1 knockdown cells (Figure 1C). Similarly, transwell invasion assays showed that AEG-1 knockdown markedly reduced the invasiveness of both Um1 and Scc25 cells (Figure 1D).

**Figure 2:**
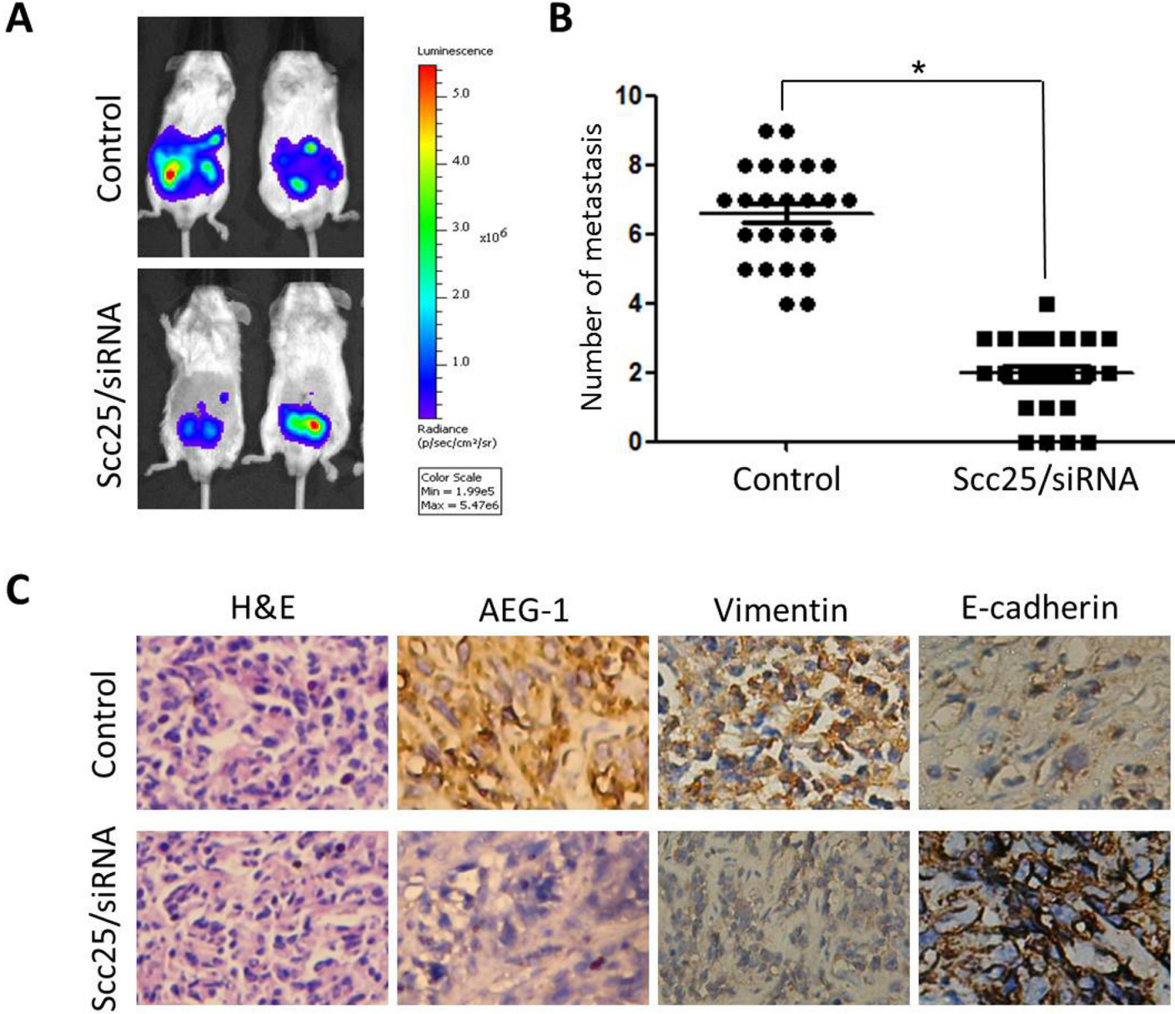
AEG-1 knockdown inhibited tumor metastasis *in vivo* **A.** Representative BLI images of athymic nude mice bearing Scc25/AEG-1-siRNA tumors with metastatic lesions. Mice (*n* = 25) were imaged 6 weeks after tumor cell injection to observe local tumor growth and metastasis. **B.** Number of metastatic nodules or distant metastasis in individual dead mice bearing control cell or Scc25/AEG-1-siRNA tumors according to H&E-staining results. **C.** AEG-1 knockdown in Scc25 cells suppressed EMT in mice. H&E staining showed primary tumors with detectable metastases in control mice and without metastases in mice bearing Scc25/AEG-1-siRNA tumors 6 weeks after injection (magnification, ×200). Immunohistochemistry revealed that AEG-1 knockdown decreased vimentin expression and increased E-cadherin staining (magnification × 200).

We next explored the effect of AEG-1 on the metastatic activity of Scc25 cells *in vivo* by subcutaneously injecting Luc-expressing Scc25 and Scc25-siRNA cells into the flank of nude mice (25 for each group). Six weeks later, Scc25-siRNA cells predominantly localized to tumor nodules in the primary injection sites, whereas the Scc25 cells formed tumors in the peritoneum cavity as well as the primary injection site. Using the Luc signal, we counted the number of metastatic nodules (Figure 2A). As shown in Figure 2B, Scc25 cells formed a greater number of abdominal metastases than Scc25-siRNA cells (6.4 ± 1.1 vs. 2.1± 0.3, *p* < 0.03, respectively). These *in vivo* results confirm that AEG-1 promotes TSCC invasion.

### AEG-1 overexpression is closely associated with EMT status in TSCC

**Figure 3:**
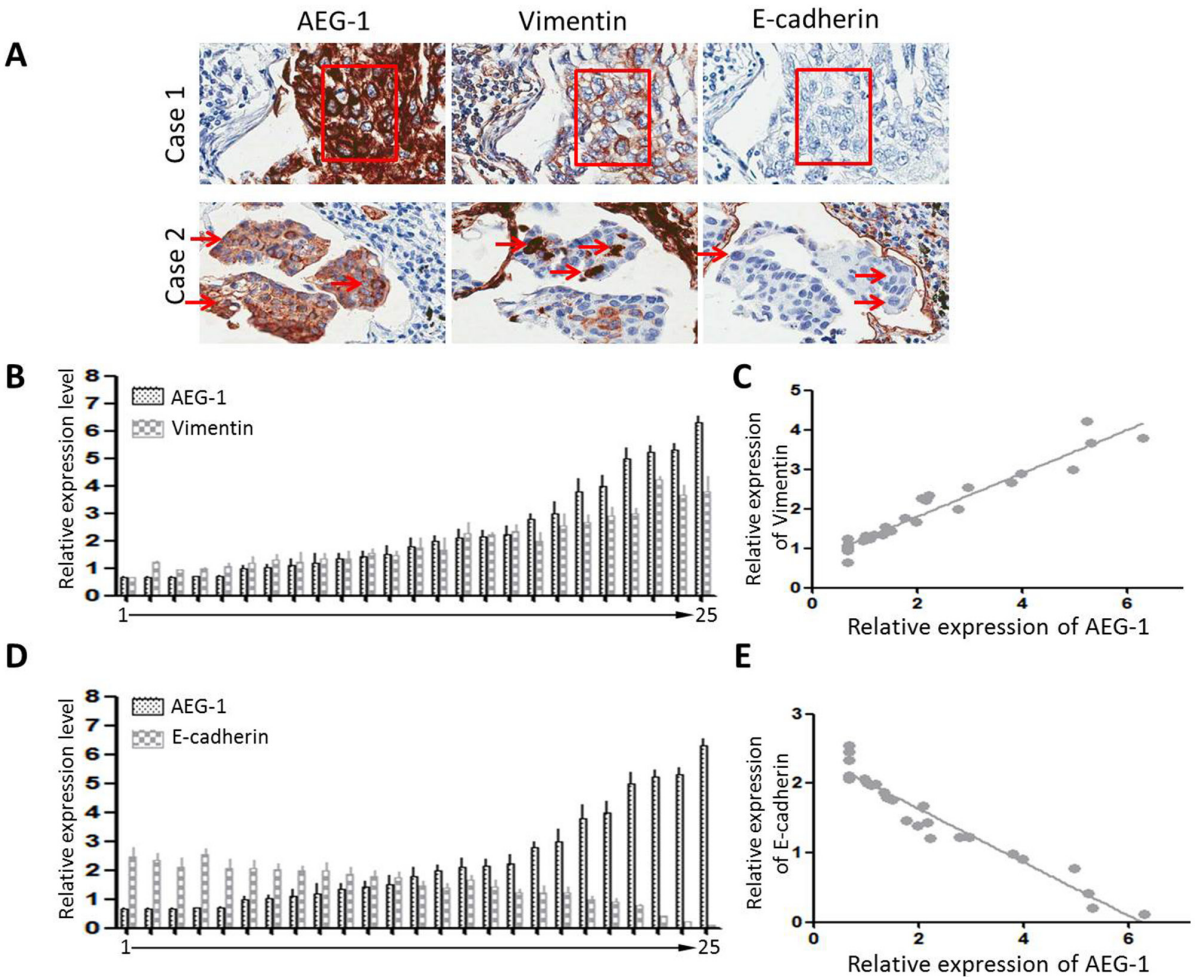
Expression of AEG-1, E-cadherin and vimentin in TSCC samples **A.** Serial sections showing two representative cases: case 1, a cluster of cells undergoing EMT; case 2, arrows indicate EMT-positive cells. These cells express high levels of AEG-1 and vimentin, but low levels of E-cadherin. **B.** and **C.** TSCC tissue samples were collected from 25 patients. For each patient, AEG-1 and vimentin were evaluated by real-time PCR and standardized against β-actin. The Pearson correlation coefficient is 0.84. **D.** and **E.** AEG-1 and E-cadherin were evaluated by real-time PCR and standardized against β-actin. The Pearson correlation coefficient is −0.91.

Immunohistochemical analysis showed that the majority of tumor cells in Scc25 xenografts exhibited stronger vimentin staining and weaker E-cadherin staining than Scc25-AEG-1-siRNA cells (Figure 2C). In clinical samples from TSCC patients, dispersed or clusters of cancer cells at the margins of the primary tumors exhibited a mesenchymal or amoeboid morphology. In addition, immunostaining showed high levels of vimentin and low levels of E-cadherin, which is characteristic of EMT, and high levels of AEG-1 (Figure 3A). The fact that AEG-1 expression was correlated positively with vimentin (*r* = 0.84) (Figure 3B and 3C) and inversely with E-cadherin (*r* = −0.91) (Figures 3D and 3E) suggests that AEG-1 might be closely associated with the EMT process.

**Figure 4:**
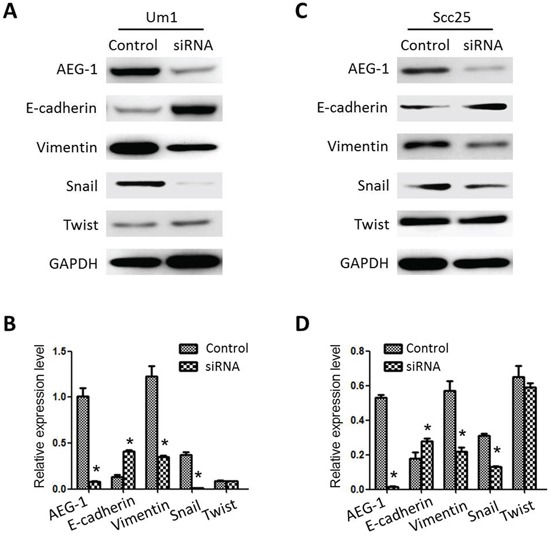
Expression characteristics of EMT-related markers in TSCC cell lines **A.** and **B.** Relative levels of AEG-1, E-cadherin, vimentin, Snail and Twist expression in Um1 cells. GAPDH served as the control. **C.** and **D.** Relative levels of AEG-1, E-cadherin, vimentin, Snail and Twist expression in Scc25 cells. GAPDH served as the control. (*P < 0.05).

We examined the effect of AEG-1 depletion on the EMT-like phenotype of the cells using Western blot. As shown in Figure 4A and 4B, expression of the mesenchymal markers vimentin and Snail was significantly lower in AEG-1-depleted Um1-siRNA cells than control cells, whereas the expression of E-cadherin, an epithelial marker, was enhanced in the Um1-siRNA clones. Similar changes of EMT markers following AEG-1 knockdown were evidently observed in Scc25-siRNA clones (Figure 4C and 4D).

### AEG-1 activates Wnt/PCP-Rho signaling in TSCC cells

**Figure 5:**
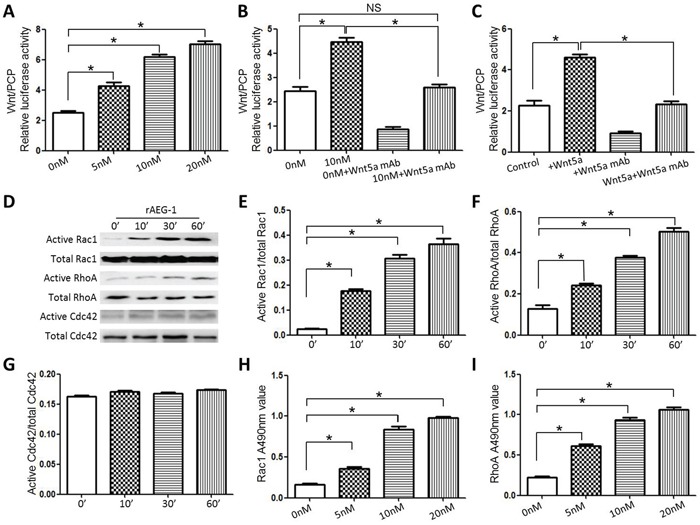
AEG-1 activated Wnt/PCP signaling in Scc25 cell lines **A.** rAEG-1 activated noncanonical Wnt/PCP signaling in TSCC cells, and the effect was dose-dependent. **B.** and **C.** The stimulatory effects of rAEG-1 (B) and Wnt5a (C) on Wnt/PCP signaling were blocked by a neutralizing anti-Wnt5a mAb. **D.** Pull-down assays of active and total Rac1, RhoA and Cdc42 in Scc25 cells treated with rAEG-1. **E–G.** Quantitative analysis of grey values obtained for active Rac1/total Rac1 ratios (E), active RhoA/total RhoA ratios (F) and active Cdc42/total Cdc42 ratios (G) using Image J software. **H.** and **I.** Rac1 G-LISA assays were used to assess the levels of GTP-bound Rac1 (H) and RhoA (I) in Scc25 cells treated with rAEG-1. (*P < 0.05).

To investigate the molecular mechanism underlying the positive impact of AEG-1 on TSCC cell migration and invasion, we carried out luciferase assays with an ATF2 reporter system. Our results demonstrated that recombinant (r)AEG-1 activated non-canonical Wnt/PCP signaling in Scc25 cells, and that the rAEG-1-induced signaling was obviously dose-dependent (Figure 5A). Moreover, the effects of rAEG-1 could be reversed by a neutralizing mAb against Wnt5a (a ligand of the noncanonical Wnt/PCP pathway) (Figure 5B). We also confirmed the effects of Wnt5a and the anti-Wnt5a mAb on Wnt/PCP signaling in Scc25 cells (Figure 5C).

**Figure 6:**
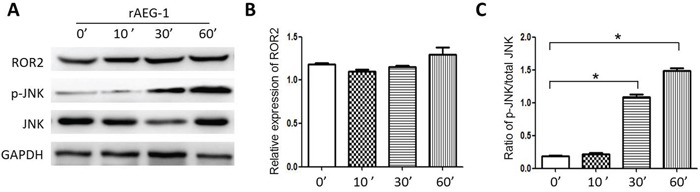
Effect of AEG-1 on ROR2 and p-JNK expression **A.** Expression of ROR2 and the phosphorylation of JNK were detected by western blotting after treatment with rAEG-1. **B.** Semi-quantitative analysis of the grey value for ROR2 obtained using Image J software at different 0, 10, 30 and 60 min. **C.** Semi-quantitative analysis of the grey value for the phospho-JNK/total JNK ratio obtained using ImageJ software (*p < 0.05).

The small Rho GTPases Rac1, RhoA and Cdc42, are key mediators in the Wnt/PCP pathway and important contributors to tumor migration and invasion. Using Rho GTPase pull-down assays, we observed that rAEG-1 promoted the activities of RhoA and Rac1 but not Cdc42 (Figure 5D–5G), and this finding confirmed by the results of GLISA assays (Figure 5H and 5I). In addition, activation (phosphorylation) JNK (c-Jun N terminal kinase), another downstream mediator in the Wnt/PCP pathway, was also enhanced by exogenous rAEG-1 (Figure 6A–6C).

### AEG-1-mediated TSCC invasion and EMT are Wnt/PCP signaling-dependent

**Figure 7:**
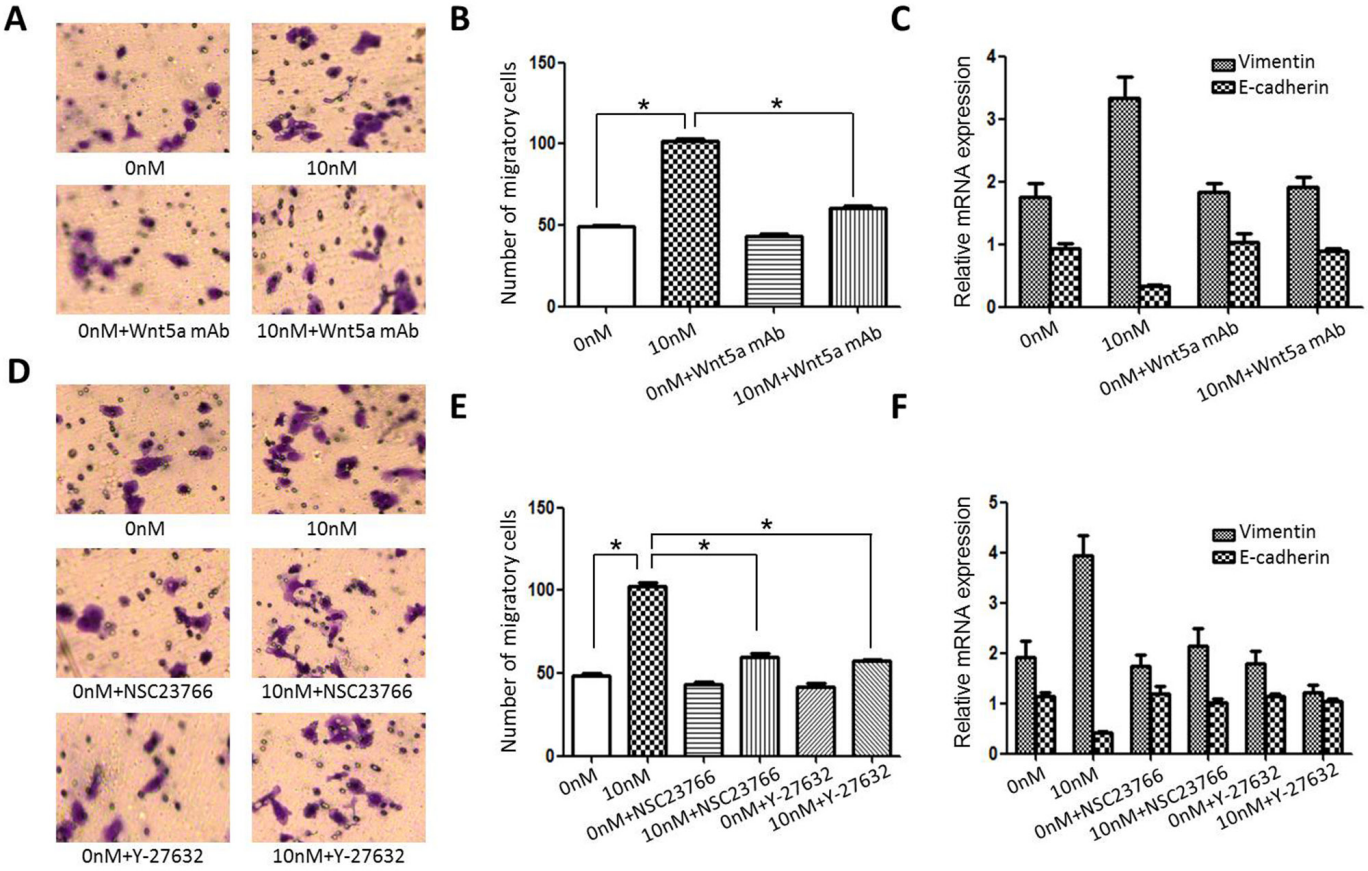
Effect of blocking Wnt/PCP signaling on Scc25 cell invasiveness **A.** The stimulatory effect of rAEG-1 on tumor invasiveness was blocked by an anti-Wnt5a neutralizing mAb. **B.** Migrated cells were counted in six randomly selected fields (original magnification: 200×). **C.** The stimulatory effect of rAEG-1 on EMT status was inhibited by an anti-Wnt5a neutralizing mAb. **D.** The stimulatory effect of rAEG-1 on tumor invasiveness was partially blocked by a Rac1 inhibitor (NSC23766) or a ROCK inhibitor (Y-27632). **E.** Migrated cells were counted in six randomly selected fields (original magnification: 200×) (*p < 0.05). **F.** The stimulatory effect of rAEG-1 on EMT was inhibited by a Rac1 inhibitor (NSC23766) or a ROCK inhibitor (Y-27632).

To determine whether AEG-1 promotes invasion and EMT through Wnt/PCP signaling, we used a neutralizing anti-Wnt5a mAb or the Wnt/PCP signaling-specific inhibitors Y-27632 and NSC23766 to suppress WNT/PCP signaling in Scc25 cells. We observed that the stimulatory effects of rAEG-1 on Scc25 cell invasion and EMT status were almost completely blocked by the anti-Wnt5a mAb (Figure 7A–7C). Similarly, Y-27632 (a ROCK inhibitor) and NSC23766 (a Rac1 inhibitor) not only inhibited the positive effect of rAEG-1 on invasion, they reduced vimentin levels and increasing E-cadherin levels in rAEG-1-treated Scc25 cells (Figure 7D–7F). Collectively, these results suggest that AEG-1 stimulates activity in a Wnt/PCP-Rho-JNK pathway, thereby promoting EMT and TSCC migration and invasion.

### Prognostic value of AEG-1 and EMT status in TSCC patients

**Figure 8:**
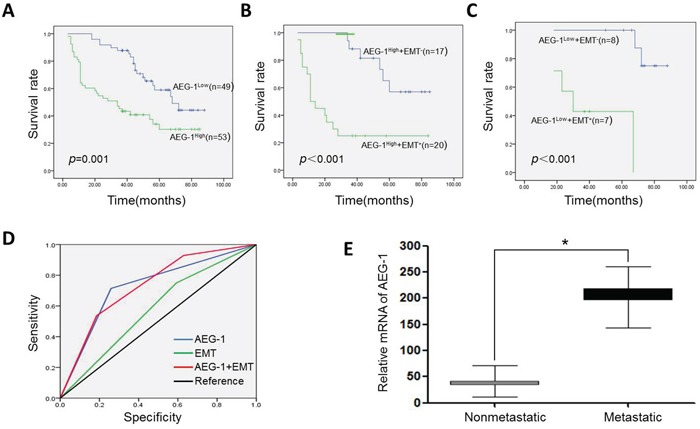
Effect of AEG-1 and EMT status on Kaplan-Meier survival curves, and their corresponding ROC analysis **A.** High AEG-1 expression predicted poor survival among TSCC patients. **B.** In the group with AEG-1-high expression, EMT(+) patients showed a poorer survival trend. Patients that were E-cadherin(−) and vimentin(+) were defined as EMT(+). Patients that were E-cadherin (+) and vimentin(−) were defined as EMT(−). **C.** In the group with AEG-1-low expression, EMT(+) patients also exhibited a poorer survival trend. **D.** The combined AEG-1 expression characteristics and EMT status had a larger area under the ROC curve than either AEG-1 level or EMT separately. **E.** AEG-1 mRNA expression was evaluated using real-time RT-PCR in metastatic TSCCs (n = 12) and those without metastasis (n = 30). AEG-1 mRNA levels were standardized as the AEG-1/β-actin mRNA ratio. (*P < 0.05).

To determine whether AEG-1 could be useful for predicting the clinical outcomes of TSCC patients, we used Kaplan-Meier survival analysis to evaluate the correlation between AEG-1 expression and prognosis among 102 TSCC patients. Survival data showed that TSCC patients whose tumors were AEG-1-high experienced shorter overall survival times than those whose tumors were AEG-1-low (*P* = 0.001). As shown in Figure 8A, the 5-year survival rate among TSCC patients expressing high levels of AEG-1 (16.98%, 95% CI: 32.04%-50.13%) was significantly lower than among patients expressing lower levels of AEG-1 (36.73%, 95% CI: 58.68%-73.08%) (Figure 8B and 8C).

We also used the receiver operating characteristic (ROC) method to evaluate how predictive of death were the AEG-1 and EMT statuses in TSCC patients. As shown in Figure 8D, the combination of AEG-1 levels and EMT status was reliably predictive of the death rate. The area under the ROC curve was 0.732 (95% CI: 0.599-0.866). By comparison, the areas under the separate ROC curves for AEG-1 levels and EMT status were 0.728 (95% CI: 0.590-0.865) and 0.579 (95% CI: 0.426-0.731), respectively (Figure 8D). Real-time PCR analysis consistently showed substantial levels of AEG-1 expression during the follow-up period in TSCC patients with distant metastasis (*n* = 12), but significantly less expression in those without metastasis (*n* = 30) (Figure 8E). These data indicate that AEG-1 up-regulation may be an indicator of distant metastasis in TSCC.

## DISCUSSION

AEG-1 is an oncoprotein that acts to modulate cell proliferation, angiogenesis, invasion and metastasis in many human malignancies [18, 19]. In the present study, we found that AEG-1 is aberrantly expressed in TSCC cells and that its knockdown significantly inhibits TSCC cell motility. Using a xenograft model, we also revealed that AEG-1 knockdown in the implanted TSCC cells substantially decreases the incidence of lymph node metastasis. This is in line with earlier studies demonstrating that AEG-1 knockdown or administration of an anti-AEG-1 antibody reduced lung metastasis by 4T1 breast cancer cells [9]. In TSCC patients, moreover, we confirmed a positive association between AEG-1 overexpression and distant metastasis. These results lend further credence to the idea that AEG-1 is a clinically relevant promoter of TSCC progression and metastasis.

In recent years, it has been well established that AEG-1 promotes tumor progression and metastasis in several malignancies, including hepatocellular carcinoma [20] and osteosarcoma [21]. However, its actions to enhance malignant phenotypes in TSCC remain poorly defined. One novel finding in this study is that AEG-1 expression is closely associated with vimentin, Snail and E-cadherin, three key EMT-related markers. EMT entails the acquisition by epithelial cells of mesenchymal characteristics, such a vimentin-cytoskeleton and an elongated fibroblast-like morphology, as well as a capacity for invasion and metastasis [22, 23]. As EMT contributes to the malignancy of TSCC [24, 25], a positive correlation between AEG-1 expression and EMT status in clinical samples may provide insight into the function of AEG-1 in TSCC progression.

Using TSCC cell lines stably expressing AEG-1-targeting siRNA, we showed that AEG-1 knockdown leads to increased expression of E-cadherin and decreased expression of vimentin which implies suppression or reversal of the EMT process [26, 27]. We also found that suppression of AEG-1 reduces Snail expression but had no effect on Twist expression. Snail directly represses E-cadherin expression by binding to the E-box elements in the E-cadherin promoter [28, 29]. These results support the hypothesis that AEG-1 contributes to the EMT process in TSCC, but the precise mechanism by which AEG-1 knockdown suppresses EMT remains unclear.

As an important oncogene, AEG-1 exerts its various effects by modulating a diverse array of signaling pathways, including those mediated via NF-κB, PI3K/Akt and Wnt/β-catenin [10, 11, 30]. Because in luciferase reporter assays expression of recombinant AEG-1 led to the dose-dependent activation of a non-canonical Wnt/PCP pathway, we suspect that Wnt/PCP contributes to AEG-1-induced tumor invasion and EMT. Consistent with that idea, the pro-invasion capacity of rAEG-1 was almost completely blocked by a neutralizing antibody against Wnt5a (a ligand of Wnt/PCP pathway [31]) or a specific inhibitor of Rac1 or ROCK (downstream mediators of Wnt/PCP signaling [32]). Importantly, similar effects on EMT status were also achieved by the interaction between rAEG-1 and Wnt/PCP signaling. As with the canonical Wnt pathway, Wnt/PCP signaling is essential for such morphogenetic events as formation of cell protrusions [33] and cell migration [34]. This pathway also carries signals from cell-surface Frizzled receptors and ROR2/RYK co-receptors to the nucleus via Rho GTPases (e.g., Rac1, RhoA and Cdc4) and JNK (c-jun-NH2-terminal kinase) [35]. Here we demonstrated that AEG-1 mediates TSCC invasion and EMT through activation of the Rho GTPases Rac1 and RhoA, and by promoting the phosphorylation (activation) of JNK. Rac1 and Cdc42 enhance actin polymerization contributing to the formation of protrusive forces [36, 37]. However, AEG-1 activates Rac1 and has no significant effect on Cdc42 activity, indicating that Rac1 mediated AEG-1-activating Wnt/PCP pathway. These results support the notion that during TSCC progression, AEG-1-overexpressing cells effectively escape from the primary lesion through induction of Wnt/PCP signaling-mediated EMT, thereby enhancing the metastatic tropism of TSCC cells.

Our findings indicate that EMT and the migratory/invasive phenotypes of AEG-1-overexpressing TSCC cells are Wnt/PCP signaling-dependent events. Furthermore, the association and evaluation of AEG-1 expression and EMT status could potentially serve as a predictive marker of the prognosis in post-operative TSCC patients.

## MATERIALS AND METHODS

### Cell culture and tissue specimen selection

The Um1 and Scc25 TSCC cell lines were maintained in Dulbecco's modified Eagle's medium (DMEM; Invitrogen, USA) supplemented with 10% calf serum (Gibco, Grand Island, NY, USA) at 37°C under a 5% CO_2_ atmosphere. GV248-AEG-1-siRNA (siRNA sequences were from Jikai, China) was transfected using Lipofectamine 2000 reagent (Invitrogen, USA). Stable monoclone was realized by ten-time diluting transfected cells in a 96-well plate. Silencing effect was verified by Western blot analysis. Control nontarget sequence was used to to exclude off-target effects of siRNA.

A total of 102 surgical specimens collected from 2003 to 2007 and coded as “squamous cell carcinoma of the tongue” were obtained consecutively from the pathology archives of the Affiliated First Hospital, Sun Yat-sen University. Their earlier pathological reports were confirmed by a senior pathologist. Follow-up data was collected through telephone interviews. The protocol for this study was approved by the Medical Ethics Committee of Sun Yat-sen University, and informed consent forms were obtained from each patient.

### Scratch wound-healing assay

Variously treated cells were grown to confluence in a 24-well plate, after which a sterile micropipette tip was then used to create a scratch on the monolayer of cells. The plates were then washed 3 times to remove cellular debris, replenished with serum-free medium, and incubated at 37°C under a 5% CO_2_ atmosphere. The width of the wound was measured under the inverted microscope at 0 h and after 72 h to assess cell migration.

### Cell invasion assay

Invasiveness was measured using 24-well BioCoat cell culture inserts (Costar, New York, NY, USA) containing a polyethylene terephthalate membrane (8-μm pores) coated with Matrigel Basement Membrane Matrix (Cultrex, MD, USA). At the end of the assay, cells that did not migrate through the pores were removed with a cotton swab. Invasiveness was determined by counting the cells that migrated to the lower side of the filter.

### Western blot

Western blotting was performed as previously described [38]. Equal quantities of cell lysate were added to SDS-PAGE gels. Blotted polyvinylidene difluoride membranes were probed with antibodies against AEG-1 (Invitrogen, USA), Snail, Twist 1, E-cadherin, vimentin, ROR2, JNK, p-JNK and GAPDH (Abcam, Cambridge, UK) in 5% milk/TBST (tris-buffered saline Tween-20) according to the manufacturer's recommendations. The bands were visualized using an ECL kit (CWBIO Technology, China).

### Quantitative real-time PCR

Q-PCR was performed in a 20-μl volume containing LightCycler FastStart DNA Master SYBR green I (Roche, USA) and 5 μl of cDNA. The relative differences between experimental groups were expressed based on cycle threshold (Ct) values. The primers used were as follows: for AEG-1, 5′-CGAGAAGCCCAAACCAAATG-3′ (sense) and 5′-TGGTGGCTGCTTTGCTGTT-3′ (antisense); for E-cadherin, 5′-TGCCCAGAAAATG AAAAAGG-3′ (sense) and 5′-GTGTATGTGGCAAT GCGTTC-3′ (antisense); and for vimentin, 5′-GA GAACTTTGCCGTTGAAGC-3′ (sense) and 5′-GCTT CCTGTAGGTGGCAATC-3′ (antisense). β-actin (primers: 5′-GCATGGGTCAGAAGGATTCCT-3′ (sense) and 5′-TCGTCCCAGTTGGTGACGAT-3′ (antisense)) was used to normalize the Q-PCR values.

### *In vivo* xenograft studies in athymic nude mice

For the xenograft model, male nude mice (about 8 weeks of age) were anesthetized with sodium pentobarbital (50 mg/kg) in a sterile environment. Scc25 (2×10^6^) or Scc25/AEG-1-siRNA cells (2×10^6^) in 50 μl of PBS were then subcutaneously injected into a flank of the mice (n=25 for the Scc25 group and Scc25/AEG-1-siRNA group, respectively) using 1-ml syringes with hypodermic needles. At selected times after injection, the mice were killed, and the tumors were excised and immersed in 10% neutral buffered formalin overnight for later immunohistochemical study. For H&E staining, deparaffinized tissue sections were stained with hematoxylin and eosin. Simultaneously, two experienced pathologists are employed to analyze H&E status of primary tumor and metastatic lymph nodes. Tumor growth was assessed using a caliper and an IVIS Imaging System (Xenogen). Living Image and Xenogen software was used to analyze the images and bioluminescent signals. All animal experiments were carried out according to protocols approved by the Medical Ethics Committee of Sun Yat-sen University.

### Immunohistochemical staining

Sections (4 μm) of paraffin-embedded samples were incubated with anti-AEG-1 (1:300) (Abcam, Cambridge, UK), anti-E-cadherin (1:200) (Abcam, Cambridge, UK) or anti-vimentin (1:250) (Abcam, Cambridge, UK) primary antibodies. We applied the known positive slice in the SP kit (Maxim-Bio, Fuzhou, China) as a positive control. Sections developed using 3, 3”-diaminobenzidine as the chromogen and hematoxylin as the counterstain. The numbers of positive cells were semi-quantitatively evaluated under a light microscope. The staining index was calculated using Aperio ImageScope software (Aperio Technologies).

### Recombinant AEG-1 protein expression, purification and verification

PCR-amplified AEG-1 ORF was inserted into the episomal expression vector pET-30a. AEG-1 was then recombinantly expressed in *E. coli* BL21 (DE3) competent cells, which were seeded on Lysogeny Broth agar containing 30 μg/ml kanamycin. After incubation for 6 days at 37°C, the culture media were collected, filtered using 0.45 μm membrane filters and applied to His-bind resin columns. The column was then washed with binding buffer until a baseline UV reading was reached, and the target proteins were eluted using elution buffer containing 500 mM imidazole. The purified fractions were further quantified using a Nanodrop 2000 spectrophotometer (Thermo Fisher Scientific) and identified using SDS-PAGE.

### Luciferase reporter gene assay

For reporter gene assays, Scc25 cells seeded into 96-well plates were co-transfected with a mixture of 200 ng of ATF2 reporter plasmid (Wnt/PCP signaling) and 5 ng of pRL-SV40 Renilla luciferase plasmid (as an internal control) using Lipofectamine 2000 (Invitrogen) and following the recommended protocol. When indicated, cells were treated with 10 nM rAEG-1 or an anti-Wnt5a neutralizing monoclonal antibody. Cell extracts were prepared 48 h after incubation, and firefly and Renilla luciferase activities were measured using the Dual-luciferase Reporter Assay System (Promega, USA) with a FB-12 luminometer (Berthold).

### GTPase pull down assay

Confluent Scc25 cells were serum-starved for 24 h and treated with 10 nM rAEG-1 for 10, 30 or 60 min. Active Rac1, RhoA and Cdc42 were pulled-down from the lysates using either the GST-PBD or GST-RBD fusion protein. The total lysates and pull-down fractions were separated by SDS-PAGE on 15% acrylamide gels. The primary antibodies used were mouse monoclonal antibodies against Rac1 (Upstate Biotechnology, 1:1500), RhoA (Santa Cruz, 1:1500) or Cdc42 (Cell Signaling Technology, 1:5000). GTP-bound GTPase was standardized against the total GTPase in the lysates.

### GLISA assay

To evaluate relative Rac1 and RhoA activity in Scc25 cells, another commercial GLISA kit (Cytoskeleton Inc. Denver, CO) was used. Overnight serum-starved Scc25 cells were treated according to operating procedure. Then, snap frozen cell lysates were thawed and added to the Rho-GTP plates. After the plates were washed, an antigen-presenting buffer was added, followed by primary and secondary antibodies. Horseradish peroxidase reagent was used to detect the reaction. The plates were analyzed promptly on a microplate spectrophotometer by measuring absorbance at 490 nm.

### Statistical analysis

Data are presented as the mean ± SEM (n=3). Statistical analyses were performed using SPSS software package (version 16.0; SPSS, Chicago, IL, USA). Student's *t*-test was used to determine the statistical significance of differences between the experimental groups. Overall survival (OS) was estimated using the Kaplan-Meier method with the log-rank test. ROC curve analysis was used to determine the cutoff value of high or low AEG-1 expression and EMT status. Values of *P* < 0.05 was considered significant.
